# Acute Kidney Injury Caused by Renin-Angiotensin System Inhibitors During Minimal Change Disease Treatment

**DOI:** 10.7759/cureus.30346

**Published:** 2022-10-16

**Authors:** Kenta Torigoe, Yuta Ikemi, Yuki Yoshida, Ryosuke Sakamoto, Ayuko Yamashita, Shinichi Abe, Kumiko Muta, Hideyuki Arai, Hiroshi Mukae, Tomoya Nishino

**Affiliations:** 1 Nephrology, Nagasaki University Hospital, Nagasaki, JPN; 2 Internal Medicine, Kusumoto Naika Clinic, Omura, JPN; 3 Respiratory Medicine, Nagasaki University Graduate School of Biomedical Sciences, Nagasaki, JPN

**Keywords:** hypoalbuminemia, primary nephrotic syndrome, renin–angiotensin system inhibitors, minimal change disease, acute kidney injury

## Abstract

A 76-year-old Japanese man with nephrotic syndrome was admitted to our department for treatment. After his admission, he was administered prednisolone (PSL) at 40 mg/day, and a percutaneous renal biopsy was performed. However, on the first day of admission, his urinary protein decreased from 5.05 g/gCr to 1.85 g/gCr. On the fourth day of admission, his urinary protein further decreased to 0.38 g/gCr and the patient developed acute kidney injury (AKI). Renin-angiotensin system (RAS) inhibitors were suspected to be the cause of AKI; therefore, they were discontinued. After the renal function improved, the urinary protein worsened again to 5.49 g/gCr. Renal pathology suggested minimal change disease (MCD); therefore, PSL was continued. The patient’s urinary protein subsequently improved and he had no renal function impairment. Minimal change disease can be complicated by AKI through intravascular volume depletion caused by high urinary protein and hypoalbuminemia. However, when MCD is complicated by RAS inhibitor-associated AKI, the urinary protein may notably decrease, and the patient may present with an atypical course of MCD-associated AKI.

## Introduction

In cases of primary nephrotic syndrome, acute kidney injury (AKI) is an important complication associated with mortality and the risk of chronic renal failure [1,2]. Minimal change disease (MCD) is most likely associated with AKI among nephrotic syndromes [3]. While the causes of AKI complicating MCD are diverse, decreased circulating blood volume due to hypoalbuminemia and subsequent tubular necrosis are important mechanisms [3]. Therefore, clinicians should carefully monitor complications such as AKI when patients with MCD present with severe urinary protein and hypoalbuminemia.

Renin-angiotensin system (RAS) inhibitors reduce proteinuria by decreasing intraglomerular pressure and are recommended as supportive therapy for nephrotic syndrome, especially in nephrotic syndrome with hypertension [4,5]. Renin-angiotensin system inhibitors are essential drugs in renal diseases with proteinuria; however, AKI is an important side effect of RAS inhibitors [6]. Herein, we report a case of MCD complicated by RAS inhibitor-induced AKI; unlike typical cases of MCD complicated by AKI, the patient showed a notable decrease in urinary protein.

## Case presentation

A 76-year-old Japanese man with nephrotic syndrome was admitted to our hospital for treatment. Approximately two weeks before admission, the patient noticed the edema in his lower legs, for which he visited his family doctor and was administered azosemide. However, he gained 4 kg and had generalized edema three days before admission. He revisited his family doctor and was referred to our hospital on the same day because he had proteinuria. He was diagnosed with nephrotic syndrome at our hospital because he had a urinary protein of 5.05 g/gCr and serum albumin (Alb) of 2.7 g/dL. He had a history of hypertension and was taking eplerenone 5 mg and olmesartan 5 mg. He had no history of abnormal urinalysis, and his renal function included creatinine (Cr) of 1.03 mg/dL and an estimated glomerular filtration rate (eGFR) of 54.2 mL/min/1.73 m^2^ four days before admission. On admission, he was 155.5 cm tall and weighed 67 kg, an increase of 3 kg from before the onset of nephrotic syndrome. His temperature was 36.6℃, heart rate was 67 beats/minute, blood pressure was 128/77 mmHg, and oxygen saturation (SpO_2_) was 98% (room air). Urinary volume was 420 mL/day. Physical examination revealed marked edema in both lower legs. Blood tests revealed decreased renal function with Cr of 1.47 mg/dL and eGFR of 36.73 mL/min/1.73 m^2^. Total protein was 5.3 g/dL, and Alb was 2.7 g/dL. A urine test revealed urinary protein of 1.85 g/gCr, and there were 50-99 red cells per high-power field, indicating hematuria and proteinuria. However, the urinary protein was lower than that before admission. The selectivity index was 0.14. Other immunological tests showed no abnormalities (Table 1).

**Table 1:** Laboratory findings on admission WBC: White blood cell, Hb: Hemoglobin, Plt: Platelet, TP: Total protein, Alb: Albumin, γ-GTP: Gamma-glutamyl transpeptidase, AST: Aspartate aminotransferase, ALT: Alanine aminotransferase, LDH: Lactate dehydrogenase, CPK: Creatine phosphokinase, BUN: Blood urea nitrogen, Cr: Creatine, eGFR: Estimated glomerular filtration rate, Na: Sodium, K: Potassium, Cl: Chloride, Ca: Calcium, P: Phosphorus, CRP: C-reactive protein, HbA1c: Hemoglobin A1c, MPO-ANCA: Myeloperoxidase anti-neutrophil cytoplasmic antibody, PR3-ANCA: Proteinase 3 anti-neutrophil cytoplasmic antibody, GBM: Glomerular basement membrane, IgA: Immunoglobulin A, IgG: Immunoglobulin G, IgE: Immunoglobulin E, IgM: Immunoglobulin M, C3: Complement component 3, C4: Complement component 4, CH50: Total hemolytic complement, HCV: Hepatitis C virus, HBs: Hepatitis B surface, HBc: Hepatitis B core, PT-INR: Prothrombin time-international normalized ratio, APTT: Activated partial thromboplastin time, HPF: High power field

Laboratory test	Value	Reference range
WBC (/μL)	4800	3300-8600
Hb (g/dL)	10.5	13.7-16.8
Plt (/μL)	154000	158000-348000
TP (g/dL)	5.3	6.6-8.1
Alb (g/dL)	2.7	4.1-5.1
γ-GTP (U/L)	28	13-64
AST (U/L)	22	13-30
ALT (U/L)	21	10-42
LDH (U/L)	203	124-222
CPK (U/L)	161	59-248
BUN (mg/dL)	59	8-20
Cr (mg/dL)	1.47	0.65-1.07
eGFR (ml/min/1.73m^2^)	36.73	>60
Na (mEq/L)	141	138-145
K (mEq/L)	4.8	3.6- 4.8
Cl (mEq/L)	110	101-108
Ca (mg/dL)	8.1	8.8-10.1
P (mg/dL)	4.7	2.7-4.6
CRP (mg/dL)	0.11	0.00-0.14
HbA1c (%)	5.7	4.9-6.0
Anti-nuclear antibody	negative	negative
MPO-ANCA (U/mL)	<1.0	<3.5
PR3-ANCA (U/mL)	<1.0	<3.5
GBM antibody (U/mL)	<0.5	<7
IgA (mg/dL)	185	93-393
IgG (mg/dL)	751	861-1747
IgM (mg/dL)	41.4	33-183
IgE (IU/mL)	71.4	<358
C3 (mg/dL)	102.9	73-183
C4 (mg/dL)	23.7	11-31
CH50 (CH50/mL)	44.6	30-46
HCV antibody	negative	negative
HBs antigen (IU/mL)	<0.05	<0.05
HBc antibody	negative	negative
PT-INR	1.03	0.80-1.20
APTT (sec)	28.3	24-34
D-dimer (μg/mL)	1.8	<1.0
Urinary protein (g/gCr)	1.85	<0.15
Urinary red blood cells (/HPF)	50-99	<5
Selectivity index	0.14	

**Figure 1:**
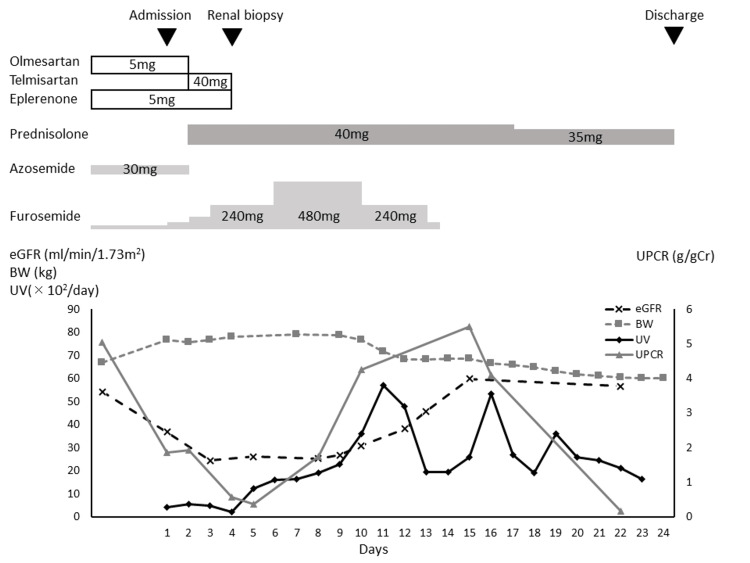
The clinical course of the patient BW: Body weight, eGFR: Estimated glomerular filtration rate, UPCR: Urine protein creatinine ratio, UV: Urine volume

**Figure 2:**
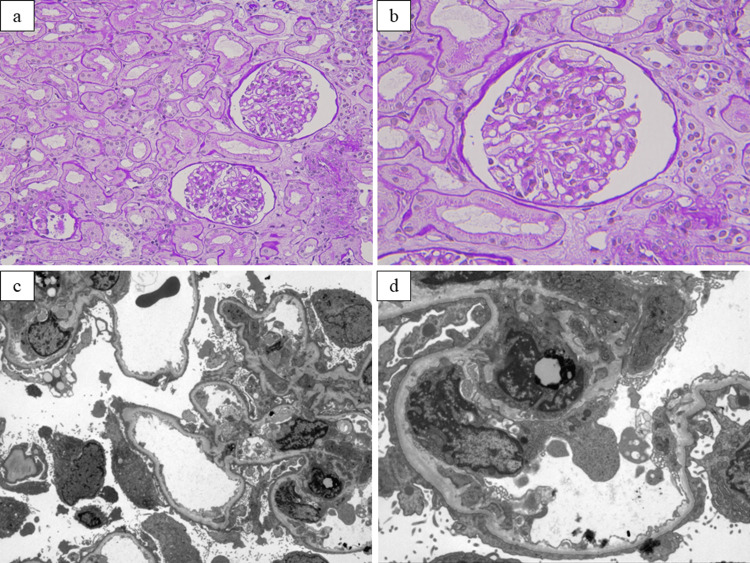
Renal pathology findings of light and electron microscopy (a) Light microscopy showed less tubulointerstitial damage (periodic acid-Schiff staining×200); (b) There was no glomerular abnormality (periodic acid-Schiff staining×400); Electron microscopy showed extensive foot process effacement: (c) original magnification 1500× (d) original magnification 4000×

**Figure 3:**
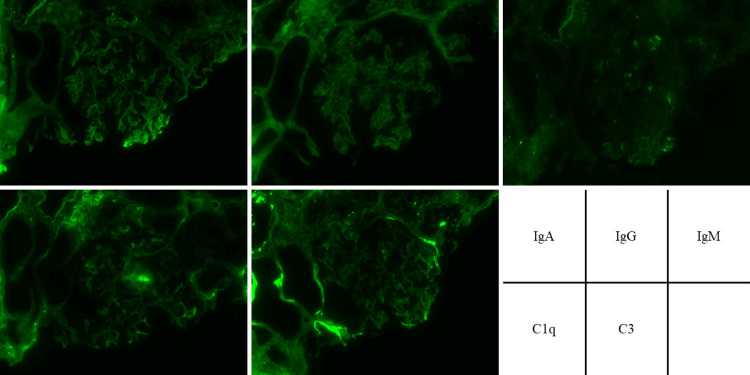
Renal pathology findings of immunofluorescence microscopy There is no deposition of IgA, IgG, IgM, C1q, and C3 in the glomerulus. IgA: Immunoglobulin A, IgG: Immunoglobulin G, IgM: Immunoglobulin M, C1q: C3: Complement component 1q, C3: Complement component 3

The clinical course of the patient is shown in Figure 1. On the second day of admission, treatment of nephrotic syndrome using prednisolone (PSL) of 40 mg/day was commenced. A renal biopsy was performed on the fourth day of admission to investigate the cause of the nephrotic syndrome. Light microscopy revealed no glomerular changes and less tubulointerstitial damage. Immunofluorescence was negative for immunoglobulin (Ig)A, IgG, IgM, complement component 1q (C1q), and complement component 3 (C3), and electron microscopy showed fusion of the foot process. These results suggested the diagnosis of MCD (Figures 2, 3). No possible secondary causes of MCD (e.g., tumor, infection, and medication) were identified; thus, MCD was diagnosed as primary MCD.

On the fourth day of admission, the patient's renal function worsened with Cr of 2.00 mg/dL and eGFR of 26.22 mL/min/1.73 m^2^; however, his urinary protein further improved to 0.38 g/gCr. Oliguria was observed at admission despite the treatment with oral diuretics, and intravenous furosemide was administered. However, the patient's weight began to increase, and respiratory failure occurred. We suspected that decreased circulating blood volume due to hypoalbuminemia caused AKI. Ultrasonography showed that the diameter of the inferior vena cava was 22/7 mm, and there was no collapse. Further, fractional excretion of urea nitrogen (FeUN) was 10.7%, suggesting prerenal kidney injury. An echographic examination of the renal arteries showed no evidence of renal artery stenosis and decreased blood flow. Therefore, the patient was suspected of having prerenal kidney injury caused by the RAS inhibitors (eplerenone and olmesartan), which he had been taking before admission, so eplerenone was discontinued. Olmesartan had been switched to telmisartan on the second day of hospitalization; thus, telmisartan was discontinued. On the sixth day of hospitalization, the patient’s urine output increased to 1600 mL/day, and his renal function, edema, and respiratory condition gradually improved. On the 15th day of hospitalization, his renal function had improved to Cr of 0.94 mg/dL and eGFR of 59.9 mL/min/1.73 m^2^; however, his urinary protein increased again to 5.49 g/gCr. The urinary protein was suspected to be associated with MCD, and PSL treatment was continued. After that, the urinary protein decreased again to 0.17 g/gCr, and the nephrotic syndrome was in complete remission on the 22nd day of hospitalization. Renal function and urine output did not decrease at that time. The nephrotic syndrome and AKI were cured, and the patient was discharged on the 24th day of hospitalization.

## Discussion

In this case, the MCD patient was complicated by RAS inhibitor-induced AKI. However, unlike a typical case of MCD complicated by AKI, the patient showed low urinary protein by RAS inhibitor-induced low intraglomerular pressure.

In cases of primary nephrotic syndrome, MCD is most likely associated with AKI, and 25% of adult cases of MCD are complicated by AKI [7]. Risk factors for AKI in MCD patients include advanced age, hypertension, urinary protein (11.6 ± 0.6 g/day), and hypoalbuminemia (1.9 ± 0.1 g/dL) [8-10]. The causes of AKI in MCD patients are diverse; however, intravascular dehydration and decreased renal blood flow associated with hypoalbuminemia, and consequent tubular necrosis are notable [3]. Therefore, in patients with severe proteinuria and hypoalbuminemia, there should be caution regarding AKI complications. In this case, at the onset of AKI, the urinary protein improved from 5.05 g/gCr to 1.85 g/gCr, and serum Alb was 2.7 g/dL, which is atypical for AKI associated with MCD. Histopathology showed no evidence of acute tubular necrosis. Fractional excretion of urea nitrogen (FeUN) was low at 10.7%, suggesting decreased renal blood flow [11]. However, in the ultrasound study, the diameter of the inferior vena cava was 22/7 mm, which did not suggest hypovolemia [12]. Furthermore, echographic examination showed no stenosis or reduced blood flow in the renal arteries, suggesting reduced blood flow at the intrarenal level.

The patient was treated for hypertension using two RAS inhibitors (eplerenone and olmesartan) before the onset of nephrotic syndrome, leading to decreased intraglomerular pressure. The concomitant use of two RAS inhibitors is a risk factor for AKI [13]. Furthermore, some reports have suggested that the use of RAS inhibitors in patients with nephrotic syndrome is associated with the risk of AKI [14,15], while another report did not suggest such a risk [16]. In this case, the nephrotic syndrome and diuretics may have further triggered the development of prerenal AKI. The use of diuretics in patients with nephrotic syndrome can cause AKI due to hypovolemia [3]. However, respiratory failure due to generalized edema and pleural effusion was observed in the present case, and the use of diuretics was unavoidable. Furthermore, aggressive infusion of fluids in cases of nephrotic syndrome with hypovolemia should be avoided because it can cause renal interstitial edema and worsen the prognosis [17]. In this case, renal interstitial edema associated with nephrotic syndrome may have been an exacerbating factor for renal dysfunction. Therefore, diuretics were continued even after the onset of AKI.

An interesting course of this case is that the urinary protein is significantly reduced due to the AKI complication caused by RAS inhibitors. In this case, the urinary protein dropped to 0.38 g/gCr in the early course of PSL administration, which at first glance seemed to indicate that PSL was effective because MCD is steroid-sensitive. However, after discontinuation of the RAS inhibitors, the urinary protein increased to 5.49 g/gCr as renal function improved. At this point, the MCD was not responsive to PSL, and the high degree of intraglomerular pressure reduction caused by the two RAS inhibitors was responsible for decreased urinary protein. Notably, because PSL reduction in MCD patients is decided based on a decrease in urinary protein [4,5], a severe decrease in urinary protein due to RAS inhibitors may lead to misjudgment of the efficacy of PSL for MCD.

## Conclusions

This report focused on a case of MCD complicated by RAS inhibitor-associated AKI. Various factors, such as diuretics, may have contributed to the AKI, but RAS inhibitors were a major contributor to AKI in this case. Renin-angiotensin system inhibitors are important renoprotective agents for nephrotic syndrome; however, caution should be exercised in cases of AKI complications, especially when patients with nephrotic syndrome are treated with two different RAS inhibitors. In addition, a severe decrease in urinary protein due to decreased intraglomerular pressure caused by RAS inhibitors may lead to misjudgment of the efficacy of PSL treatment for MCD. Therefore, careful monitoring of AKI complications and considering temporary dose reduction or discontinuation is important, especially when patients treated with two RAS inhibitors develop nephrotic syndrome.
